# LC-MS/MS Analysis and Hepatoprotective Activity of Artichoke (*Cynara scolymus* L.) Leaves Extract against High Fat Diet-Induced Obesity in Rats

**DOI:** 10.1155/2019/4851279

**Published:** 2019-05-23

**Authors:** Maryem Ben Salem, Kamilia Ksouda, Raouia Dhouibi, Slim Charfi, Mouna Turki, Serria Hammami, Fatma Ayedi, Zouheir Sahnoun, Khaled Mounir Zeghal, Hanen Affes

**Affiliations:** ^1^Laboratory of Pharmacology, Faculty of Medicine, University of Sfax, Tunisia; ^2^Laboratory of Anatomopathology, CHU Habib Bourguiba, University of Sfax, Sfax, Tunisia; ^3^Biochemistry Laboratory, CHU Habib Bourguiba, University of Sfax, Tunisia

## Abstract

*Cynara scolymus* L. (Artichoke) has been used for the treatment of metabolic disorders. The purpose of the present study was to investigate the hepatoprotective effect of* Cynara scolymus *leaves extract against a high fat diet (HFD) induced rats. This study investigated the most abundant phenolic compounds rich* Cynara scolymus* leaves extract and it is antihypercholesterolemic and antioxidative effects* in vivo*. The hypercaloric high fat diet (HFD) was treated with 200 mg/kg and 400 mg/kg of ethanol extract (EEA) from leaves of* Cynara* and atorvastatin (ATOR) (10 mg/kg/day) during an 8-week period. Lipid profile was measured and oxidative stress systematic in hepatic tissue was determined. Our data revealed that HFD-induced hepatic dysfunction manifested by significant abnormal levels of AST, ALT, ALP, LDH, and OCT was accompanied by increasing levels of oxidative stress biomarker (ROS, MDA, and AOPP) while decreasing in antioxidant status. Coadministration of EEA significantly reduced serum lipid profile and hepatic disorders which was confirmed to be histological by reducing the fatty liver deposition in hepatic lobule. These findings suggest that* Cynara* leaves exert antiobesity and antioxidant liver effects in HFD-induced obese rats.

## 1. Introduction

Nowadays, many researchers proved that obesity was a chronic metabolic disease. This explained the imbalance between the energy expenditure and intake, and it is caused seriously by increase of fat mass and lipid disposition in blood [1]. Kuboota et al. [2] and Holland et al. [3] reported that obesity is correlated with many metabolic diseases such as hypercholesterolemia, diabetes, and cardiovascular disease. According to Park et al. [4], obesity is the most consequence of liver damage, so we necessarily research therapeutic files such as herbs to advance the adequacy of the risk correlated with obesity [5, 6]. Among the medicinal plants identified from Mediterranean area (North Africa and southern Europe), there was* Cynara scolymus* (Asteraceae). According to Lattanzio et al. [7], this medicinal plant is cultivated almost around the world, because of its beneficial nutrition effects like cooking and medicinal properties. From traditional therapy,* Cynara *extract has been used as a drug in the treatment of several diseases effects of the biliary tract, digestive action, scurvy, and anemia and also has an antiartherosclerotic effect [8, 9]. Bonomi [10] explained that* Cynara* leaves extracts have been reported by many studies that it can be used alone or in association with other medicinal plants, to prepare herbal teas or medicinal plant-based capsule, according to Bonomi. Among the constituents of* Cynara* extract was the active compounds (polyphenols), which exhibited the potential antioxidants properties [11]. Many researchers reported that the treatment with various phenolic compounds achieved controlling the levels of lipid profile in HFD-fed rats [12, 13]. Yet, there is a great data available on* in vivo* research of* Cynara* leaves extract included liver complication. For that reason, we seriously plan our study to think of the greatest treatment with* Cynara* leaves extract on hepatic dysfunction and oxidative status on a model of HFD rats.

## 2. Materials and Methods

### 2.1. Preparation of* C. scolymus* Leaves Extract

Leaves around the stems of* C. scolymus* were obtained and cut into smaller pieces and then dried at room temperature under shade in order to obtain powder and to be subjected to extraction.

The protocol of extraction mentioned by 200 grams of powder leaves was extracted with different solvents and different polarities (1 L ×72 h): hexane, ethyl acetate, butanol, 75% v/v (ethanol/H_2_O), and water.

All extracts were filtered, evaporated, and removed pressure using a Rotovapor at 40°C and lyophilized by freeze-dryer (Alpha 1–2 LD plus Martin Christ®) to determine the weight of each extract and then stored at 4°C until analysis.

### 2.2. Determination of Total Phenol Content

Total phenol content of* Cynara* leaves extracts was determined using the method of Folin Ciocalteu by Fawole et al. [14]. The mixture contained 1 mL of Folin Ciocalteu reagent, 10 ml of NaCO_3_, and 1 mL of each extract. Then, the absorbance of each mixture was measured at 750 nm after 30 min. The total phenol content was expressed in terms of Gallic acid equivalent (mg of GAE/g of extract).

### 2.3. LC-MS/MS Analysis

The antioxidant compound rich EEA from leaves of* Cynara* was identified by LC-MS/MS analysis which composed an Agilent 1100 LC system (Agilent Technologies, Santa Clara, CA) containing degasser, binary pump, autosampler, and column heater. The column outlet was coupled to an Agilent MSD Ion Trap XCT mass spectrometer (Agilent Technologies, Santa Clara, CA) equipped with an ESI ion source.

The personal computer with Data Analysis software (Chemstations) evaluated data acquisition and mass spectrometric. For the chromatographic separation, we used a Zorbax 300 A° Extend-C-18 Column (2.1 150 mm; Phenomenex UK, Macclesfield, UK). The column was mixed by 95% solvent A (0.1% formic acid in water) and 5% solvent B (0.1% formic acid in acetonitrile) for 1 min, followed by an 11 min step gradient (5% B to 100% B); then it was kept for 4 min with 100% B. In the end, the elution was completed with a linear gradient from 100% to 5% B for 2 min. The flow rate was adjusted at 200 ml/ min and the volume was injected at 5 ml. The following parameters were regulated during all MS experiments: the polarity was charged with positive ion, the capillary voltage was established to 3.5 kV, the drying temperature was fixed to 350°C, the nebulizer pressure was maintained to 40 psi, and the drying gas flow was measured to 10 L/min. Moreover, the maximum accumulation time was fixed to 50 ms, the scan speed was 26 000 m/z/s (ultra scan mode) and the time of fragmentation was 30 ms.

The phenolic compounds were identified using a combination of two analytic methods: high-performance liquid chromatography (HPLC) with diode array detection and liquid chromatography with atmospheric pressure chemical ionization mass spectrometry (ESI- LC/MS/MS) on the basis of their ultraviolet (UV) spectra, mass spectra.

The mass spectra results were compared with those of available authentic standards.

### 2.4. Determination of Lipase Activity* In Vitro*

According to Nikai et al. [15], pancreatic lipase activity was measured using 4- methylumbelliferyl oleate (4-MU oleate) as a substrate. The mixture containing 25 *µ*L of each extract dissolved in DMSO and 50 *µ*L of 0.1 mM 4-MU solution dissolved in a buffer (13 mM Tris-HCl, 150 mM NaCl ) and 1.3 mM CaCl_2_ (pH 8.0) was mixed in the well of a microtiter plate and then 25 *µ*L of the lipase solution (50 U/mL) in the above buffer was added in order to start the enzyme reaction. After incubation at 25°C for 30 min, 0.1 mL of 0.1 M sodium citrate (pH 4.2) was added to block the reaction. The amount of 4-methylumbelliferone released by lipase was measured with a fluorometric microplate reader (Fluoroskan Ascent C LabSystems, Inc.) at an excitation wavelength of 360 nm with a tolerance of ±40 nm and an emission wavelength of 460 nm with a tolerance of ± 20 nm.

Orlistat is used as a positive control. PI: percentage of lipase pancreatic activity inhibition.

(1) $$\begin{array}{c} PI=\frac{Absorbance\;control-Absorbance\;test}{Absorbance\;control}*100 \end{array}$$

### 2.5. Animals and Diet

Thirty Wistar female rats, weighing 180 ± 2g, were collected from the Tunisian Pharmaceutical Industries (SIPHAT, Tunisia). The environmental conditions of animals were kept in a controlled room (60% humidity, 25°C, and 12-h light-dark cycle) in the laboratory of Animal Ecophysiology of Sfax City, Tunisia. All these animal studies have occurred in this study guided by the International Guidelines for Animal Care and were accredited by the Tunisian Ethics Committee of the University of Sfax (Sfax, Tunisia).

Before animal experimentation, all animal were kept to acclimate for one week and fed on a standard diet (corn, soy, and vitamins) supplied by the Company of Animals Nutrition, Sfax, Tunisia.

### 2.6. Experimental Design

The creation of the model of high fat diet (HFD) was composed of 79.9% normal diet, 10% sheep fats, and 0.1% cholic acid. 30 Wistar rats were divided into 5 groups (n= 6 six):

Groups I: (C). Control rats fed on a standard diet.

Groups II: (HFD). Rats fed on the high fat diet, for 8 months.

Groups III: HFD + ATOR. Rates fed on HFD and treated with 10 mg/kg /bw a commercial drug ATOR (atorvastatin) by gastric gavages route in a volume of 1 ml during 2 months daily [16].

Groups IV: HFD+ EEA (200mg/kg/bw). Rats fed on HFD and treated with ethanol extract from leaves of* Cynara scolymus* at doses 200mg/kg/bw by gastric gavages route in a volume of 1 ml during 2 months daily [17].

Groups VI: HFD+ EEA (400mg/kg/bw). Rats fed on HFD and treated with ethanol extract from leaves of* Cynara scolymus* at doses 400mg/kg/bw by gastric gavages route in a volume of 1 ml during 2 months daily [17].

### 2.7. Measurement of Body Weights and Relative Organ Weights

Body weight was measured weekly during 2 months of each group and relative liver weight after sacrifice in the experiment period regularly.

### 2.8. Biochemical Analysis

The animal was sacrificed by decapitation and the trunk blood was collected. The serum of each rat was obtained by centrifugation (3000*∗*g, 15 min, 4°C) and stored at -20°C until biochemical analysis. The analyses of serum lipase and lipid profile (Triglycerides (TG), total cholesterol (T-Ch), and high-density lipoprotein cholesterol (HDL-C)) using the corresponding commercial kits (Biolabs, France) on an automatic biochemistry analyzer at the biochemical laboratory of CHU Habib Bourguiba Hospital of Sfax.

Low-density lipoprotein cholesterol concentration (LDL-c) was determined by Friedewald et al. formula [18].

Serum levels of aspartate aminotransferase (AST), alanine aminotransferase (ALT), alkaline phosphatase (ALP), and lactate dehydrogenase (LDH) were measured in frozen aliquots of serum by standardized enzymatic procedures using commercial kits from (Biolabs, France) on an automatic biochemistry analyzer at the biochemical laboratory of CHU, Habib Bourguiba Hospital of Sfax. For the ornithine carbamoyltransferase (OCT) activity was determined by the colorimetric spectrophotometric methods [19].

### 2.9. Determination of Hematological Parameters

Hematological parameters including red blood cells (RBC), hemoglobin (Hb), mean corpuscular volume (MCV), mean corpuscular hemoglobin (MCH), mean corpuscular hemoglobin concentration (MCHC), white blood cell (WBC), and platelets were measured by Horiba ABX 80 Diagnostics (ABX pentra Montpellier, France).

### 2.10. Determination of Liver Oxidative Stress Markers

Oxidative stress was estimated through the following parameters: the extent of lipid peroxidation by measuring the thiobarbituric acid reactive substances (TBARS) in terms of malondialdehyde (MDA) formation according to Draper and Hadley [20] and expressed as nmol MDA/mg protein and the protein oxidation by measuring the advanced oxidation protein (AOPP) by Kayali et al. [21] method and expressed as nmol/mg protein.

### 2.11. Determination of Non- Enzymatic and Enzymatic Antioxidants Systems

Superoxide dismutase (SOD) activity was measured by Beyer and Fridovich [22] method and expressed as U/mg protein. Glutathione (GPx) activity was determined by the method described by Pagila and Valentine [23] and expressed as mmoles GSS/(min_mg protein). Reduced glutathione (GSH) activity was estimated according to the described method by Carlberg and Mannervik [24] and expressed as ug GSH/mg protein. The protein level was evaluated by Lowry et al. [25] using bovine serum albumin as the standard at 660 nm.

### 2.12. Determination of ROS Production

According to Gupta et al. [26] methods, the reactive oxygen species formation (ROS) in liver tissue was described with some modifications. Liver samples (200 mg) were homogenized in ice-cold Tris-HCl buffer (40 mM, pH 7.4) (1:10 w/v). Then, 100 mL of samples of tissue homogenate were mixed with Tris-HCl buffer (1 mL) and 5 mL of 20,70-dichlorofluorescein diacetate (10 mM) (Sigma-Aldrich, St. Louis, MO). The mixture then was incubated for 30 min in 37°C. After the incubation, the fluorescence intensity of the samples was assessed using a FLUOstar Omega multifunctional microplate reader (*λ*_excitation_ 485 nm and *λ*_excitation_ 535 nm).

### 2.13. Histopathological Liver Analysis

After the sacrifice of experimental rats, a small portion of each liver tissue of each rat was eliminated and fixed in 10% formaldehyde solution. Each washed tissues was dehydrated in increasing gradient of ethanol and finally cleared into toluene. The liver tissues were then embedded in molten paraffin wax. Sections were cut at 5 mm thickness and stained with hematoxylin and eosin (H&E).

## 3. Statistical Analysis

Data are expressed as mean ± standard deviation (mean ± SD). The one-way analysis of variance (ANOVA) and the Tukey post hoc test were performed on the data for intergroup comparisons. Database management and statistical analysis were performed using SPSS (SPSS Inc., Chicago, IL) statistical software package. The nominal statistical significance level was set at 0.05.

## 4. Results

### 4.1. Determination of Total Phenol Content

**Figure 1:**
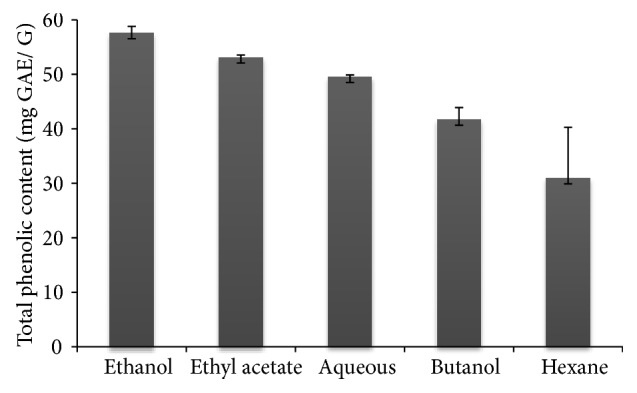
Total phenolic content of various extracts from leaves of* Cynara scolymus*. Values are given as means ± SD (n = 3).

Figure 1 showed the highest content of total phenol content was obtained using 75% of ethanol extract from leaves of* C. scolymus* as the extraction solvent and corresponded to 54.54 ± 1.26 mg GAE/g dry extract, followed by ethyl acetate, aqueous, and butanol extracts, while the hexane extract was the lowest in the total phenol content (30.91 ± 9.36 mg GAE/ g dry extract).

### 4.2. LC-MS/MS Analysis

**Figure 2:**
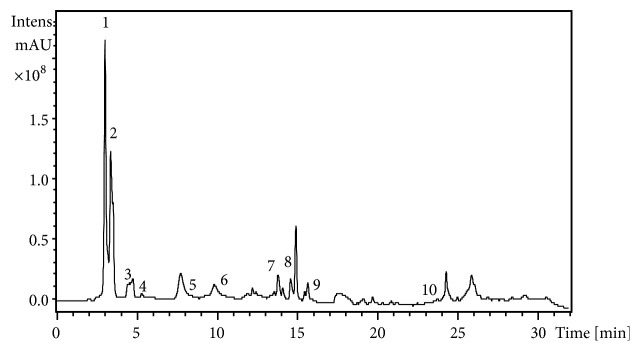
LC-MS/MS chromatogram of ethanol extract from leaves of* Cynara scolymus* at two different wavelengths (200 and 700 nm).

From the HPLC chromatogram of the EEA from leaves of* Cynara scolymus*, ten phenolic components were identified (Figure 2).  Table 1 showed the retention times, UV max, the mode (+ or −) as well as the base peak (MS), and fragment ion (MS2). In the analysis of peak 1 by LC-MS/MS, a positive molecular ion at [MS+H]+ at m/z 122.4 as well as a fragment ion at m/z of 122.3, corresponding to salicylic acid-o-hexoside, was observed. Peak 2 exhibited from the same analysis a negative molecular ion [MS−H]- at m/z 117.2 as well as a fragment ion at an m/z 100, corresponding to chlorogenic acid. Peak 3 had a negative molecular ion [MS−H]- at m/z of 112 and three negative fragment ions at m/z of 214.8, 178.8, and 352.8 corresponding to a 3-mono-o-caffeoylquinic acid. Peak 4 exhibited a negative molecular ion at [MS−H]- at m/z 141 and the negative fragment ion at m/z of 265.9 which correspond to apigenin-7-glucoside. Peak 6 revealed a negative molecular ion [MS−H]- at m/z 275 and three negative fragment ions at m/z of 529.7, 350.8, and 284.7. The corresponding compound was identified as luteolin-7-o-rutinoside. Compounds in peak 7 had a negative molecular ion at m/z 327.1 and fragment at m/z of 170.9; this is suggestive of a dihydroxypropiophend-hexoside compound. The kaempferol 3-o-rutinoside was identified in peak 8 with a negative molecular ion at [MS−H]- at m/z of 395 and fragmentation products at m/z of 529.7, 288.9, and 307. Peak 9 revealed a negative molecular ion at [MS-H]- at m/z of 431 and three negative fragment ions at m/z of 434.7, 294.9, and 344.8; the corresponding compound was identified as quercetin-o-pentoside. Compounds in peak 10 had a negative molecular ion at m/z 623 and fragment at m/z of 255.1; this is suggestive as Caffeic acid compound. In the analysis of compound in peak 5 with referring to standards and literature, it was not well identified.

### 4.3. Lipase Pancreatic Inhibition Activity

Table 2 showed that EEA presented a great pancreatic lipase inhibition with an IC50 value of 61.50 *µ*g/mL as compared to Orlistat specific inhibitor (IC50 = 17.76 *µ*g/mL). The potential in* in vitro* inhibitory effect of lipase was observed to EEA compared to the other extracts that leads us to apply it for* in vivo* investigation.

### 4.4. Evaluation of Body Absolute and Relative Organ Weights

**Figure 3:**
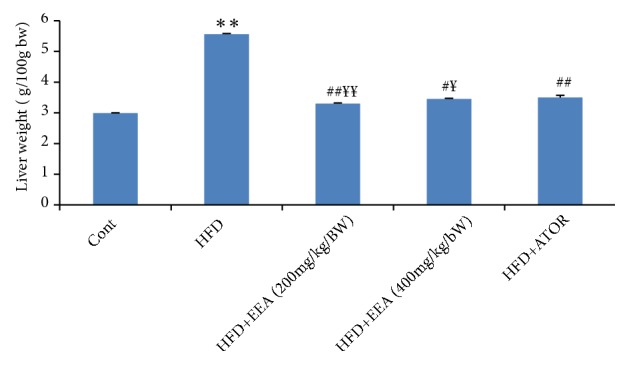
Effect of* C. scolymus* leaves extract on the liver weight of control and HFD groups. HFD groups were orally administered with ethanol extract (EEA) from leaves of* C. scolymus* and atorvastatin (ATOR) at the doses mentioned earlier for 60 days. Values are given as means ± SD (n = 6). *∗∗*p ≤ 0.01 was considered significant compared to control groups; ^#^ p ≤ 0.05; ^##^ p ≤ 0.01 were considered significant compared to HFD groups; ^¥^p ≤ 0.05; ^¥¥^ p ≤ 0.01 were considered significant compared to HFD groups treated with atorvastatin.

HFD groups showed a significant increase in body weight and liver relative weight (p<0.001) compared with the control groups, while HFD groups treated with EEA at doses (200-400 mg/kg/bw) observed a significant decrease (p<0.001) when compared to HFD groups (Table 3, Figure 3).

### 4.5. Effect of a High Fat Diet on Lipase Pancreatic in Serum

**Figure 4:**
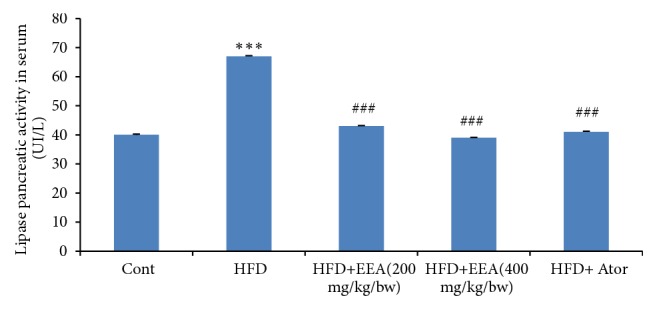
Effect of treatment with* C. scolymus* leaves extract on lipase activity in serum of control and HFD groups. HFD groups were orally administered with ethanol extract from leaves of* C. scolymus* and atorvastatin (ATOR) at the doses mentioned earlier for 60 days. Values are given as means ± SD (n = 6). *∗∗∗*p ≤ 0.001 was considered significant compared to control groups; ^###^ p ≤ 0.001 was considered significant compared to HFD groups.

Our results indicated that treatment with HFD for 8 months showed a significantly elevated (p<0.001) in lipase pancreatic activity in serum by 75 % in compared with control groups, while the treatment with EEA from leaves of* C. scolymus* and ATOR decreased significantly (p<0.001) these levels by 56 % in serum compared with HFD groups (Figure 4).

### 4.6. Effect of a High Fat Diet on Serum Lipid Profile

The effect of HFD on serum biochemical was shown in Table 4. The levels of TG, TC, and LDL-c showed a significant (p<0.001) increase by 46.06 %, 69.36 %, and 66.66 % as compared to control groups. Meanwhile, the serum level of HDL-c significantly (p<0.001) decreased by 59% in HFD groups.

The administration of EEA (200-400 mg/kg/bw) restored normal levels of TC by 45.67 %, 67.78 %, and 36.22 %, TG by 54.92 %, 68.08 %, and 60.11 %, and LDL-c by 27.27 %, 33.33 %, and 44.44 % (p<0.001) and caused significant increase in serum HDL-c level by 44.27 %, 60.79 %, and 38.76 % (p<0.001) compared to HFD groups.

Our results indicated that HFD for a duration of 8 months has significantly increased the risk of atherosclerosis indicated by the atherogenic index of plasma (AIP) and Castelli's risk index-I (CRI-I). However the therapy with EEA (200-400mg/kg/bw) significantly (p<0.001) decreased the AIP and (CRI-I) levels compared to HFD groups.

### 4.7. Effect of* Cynara scolymus* Leaves Extract in Hepatic Dysfunction Enzymes

HFD caused severe hepatotoxicity as evidenced by a significant elevation (p<0.001) of serum AST, ALT, LDH, ALP, and OCT levels, compared to control groups. However, the coadministration of EEA (200-400mg/kg/bw) and ATOR completely recovered the impaired liver functions by decreasing hepatic parameters compared with HFD groups (Table 5).

### 4.8. Effect of* Cynara scolymus* Leaves Extract on Hematological Parameters

The results indicated that, compared to control groups, there was a significant decrease in the levels of RBC, Hb, MCV, MCH, MCHC, WBC, and platelets observed in HFD groups. However, these hematological markers were considerably increased to near normal values after the treatment with EEA from leaves of* C. scolymus* (200-400 mg/kg/bw) (p < 0.001) compared with HFD groups (Table 6).

### 4.9. Effect of* Cynara scolymus* Leaves Extract in Oxidative Stress Status

HFD has significantly (p < 0.001) increased TBARS levels by 52.29 %, AOPP levels by 55.58 %, and liver ROS intracellular production by 53.33 % as compared with HFD groups (Table 7). After all, treatment with EEA at both doses (200-400mg/kg/bw) in HFD rats has improved the antioxidant capacities significantly (p< 0.001) in the liver by increasing SOD, GP-x, and GSH activities as compared to HFD groups.

### 4.10. Histopathological Analysis of Liver

**Figure 5:**
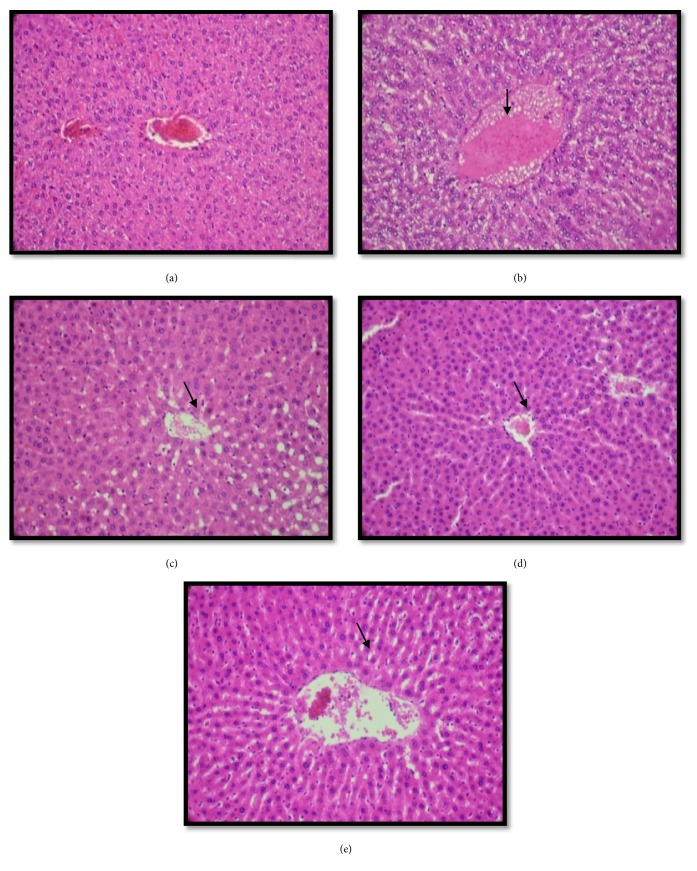
Histopathological examination of liver in experimental groups of rats. (a) represents a normal hepatic lobules of the control groups; (b) represents histopathological investigation of liver tissue in rats fed on HFD groups which showed severe macrovascular fatty changes distributed throughout the liver lobules; (c and d) represent HFD+ EEA (200- 400mg/kg/bw) groups, which showed a reduction in accumulation of fatty throughout the liver lobules; (e) represents HFD+ ATOR groups in which a potential protective action was shown (H&E × 400).

To approve our results on the biochemical analysis, we examined the pathologic changes in liver microscopically. Our study revealed that the histological findings in the liver indicated the presence of lipid accumulation in hepatic lobule in the case of the HFD groups, indicated by arrows (Figure 5(b)). However, the treatment with EEA from leaves of* C. scolymus* (200-400 mg/kg/bw) and ATOR showed a protective effect in the form of the liver histoarchitecture (Figures 5(c), 5(d), and 5(e)).

## 5. Discussion

One of the most chronic metabolic diseases was obesity, including severe human complication. It is closely related to type 2 diabetes, hypertension and cardiovascular disease according to Wilis et al. [27], cancer, respiratory complications, and osteoarthritis [28], which has been increased at an alarming percentage around the world.

According to Mnafgui et al. [29], the detection of antiobesity drugs without undesirable side effect has become a big problem. Nowadays, the medicinal plant has been used as a complementary treatment, in order to decrease the undesirable effect of drugs treated by patients.

This research was carried out to appreciate the potentiality effect of* C. scolymus* leaves extract on obese rat induced HFD and to assess its impact therapeutic about obesity.

Chakrabarti [30] demonstrated that the use of safer lipase inhibitors is considered as an efficacious therapeutic approach to overcome obesity and hyperlipidemia by the delay of the digestion and absorption of fat.

It is known that the major role of pancreatic lipase is the digestion of fats by dividing dietary triglycerides into monoacylglycerides and free fatty acids and then is the absorption by enterocytes. Among the drugs used for the prevention of obesity and hyperlipidemia by inhibition of the pancreatic lipase activity by the Orlistat [31], from our results, the pancreatic lipase activity was inhibited by EEA by hydrolyzing of dietary triglycerides nonabsorbable into monoglycerides and free fatty acids absorbable by the intestine. Our results were consistent with the observation by Mnafgui et al. [29]. Accordingly, we can explore the mentioned ethanol extract* in vivo*. The inhibition of pancreatic lipase activity was one of the solutions to avoid obesity complication [32]. The present research evidenced that EEA inhibited lipase activity in the plasma in HFD groups, which make a decrease in serum T-Ch, LDL-c, and TG levels as well as a significant reduction in the calculated atherogenic index (AI). Consequently, it showed an increase in HDL-c and a decrease in body and liver weight like antiobesity action [33]. These results were confirmed by histological findings which proved a fatty disposition throughout the liver lobules. So it is clear that the administration of EEA from leaves of* C. scolymus *for two months caused a suppressive effect on fat accumulation in all experimental groups.

These results were confirmed to Abdel Magied et al. [34] who showed the strongest hypotriglyceridemic and hypocholesterolemic effects of* C. scolymus* leaves extract from Egypt; moreover, this study showed the hypocholesterolemic effect of aqueous extract from leaves of* C. scolymus* at doses (150, 300, and 600 mg/kg bw) for one month of treatment, similar to that reported by Küskü-Kiraz et al. [35] who revealed a significant decrease in Ch-T and TG level in* C. scolymus* leaves extract at doses of 1,5mg/kg bw for two weeks in hypercholesterolemic diet rats. Meanwhile, any reduction was observed in LDL- c, TG, and VLDL-c level with a hydroalcoholic extract from leaves of* C. scolymus* at doses of 100-400 mg/kg/bw for two weeks of treatment.

The efficacious hypolipidemia effect of EEA might be associated with a high content of active compounds richness* Cynara* leaves such as phenols, flavonoids, and tannins. Among these active constituents identified by LC-MS/MS, it was mentioned that cynarin and luteolin play a crucial role in inhibiting cholesterol and triglycerides synthesis according to Ben Salem et al. [36]. Some studies showed that luteolin compound could inhibit 60% of cholesterol synthesis in the digestive tract, by regulation of HMG-CoA reductase activity [9, 37]. Moreover, Qiang et al. [38] mentioned that* C. scolymus* leaves extract is able to diminish hypercholesterolemia by increasing the fecal excretion of bile acids and by inhibition of HMG-CoA reductase activity, following a significant LDL-c decreasing effect [39].

Our results were in accordance with the results of Kucukgergin et al. [40] who reported that* Cynara* leaves extract treatment for hypercholesterolemic rats was useful for decreasing serum cholesterol and triglycerides levels of rats. On the other hand,* Cynara* extract has been proposed to be antiatherogenic, due to its lipid-reducing and antioxidant effect [41, 42].

According to Gebhardt [8], it was reported the hepatoprotective effect of* Cynara* extract by reducing the cholesterol biosynthesis and the oxidation of LDL [41, 43, 44]. Among the biological activities of* C. scolymus*, the antimicrobial properties in the gut disorder the intestinal microflora; this allows affecting the absorption of cholesterol [45]. Moreover, Brown and Rice-Evans [46] state that chlorogenic acid and luteolin may prevent atherosclerosis disease by inhibiting LDL oxidation.

The results of the biochemical analysis were confirmed by histopathological of liver tissue which showed that EEA at doses 200 mg/kg bw-400 mg/kg bw from leaves of* C. scolymus* treated HFD revealed a reduction in fatty infiltration in the liver. From these results, we can conclude that* Cynara* leaves extract may be useful for the preventive treatment of hypercholesterolemia.

Moreover, many markers proposed include mitochondrial markers, such as ornithine carbamoyl transferase, since this marker has been considered less sensitive in the detection of cellular damage than cytosolic markers such as ALT and AST [47]. However, Murayama et al. [48] showed that OCT marker was recently proved to be more sensitive than ALT and AST in the detection of hepatotoxicities induced by several toxicants. Therefore, to evaluate better the hepatotoxicity in HFD model, ALT, AST, ALP, LDH, and OCT enzymes are analyzed, and their elevation in circulation suggests significant hepatocellular damage [49, 50].

Our results revealed that EEA treatment significantly reduced these hepatic enzymatic; therefore the extract ameliorated the potential liver damage caused by HFD.

These results are in accordance with the results of Raida et al. [32] and Ben Abdallah et al. [51]. Many researchers showed the hepatoprotective effect of* C. scolymus* leaves extract, related to its active compounds. However, more researches were needed to fully explain the hepatoprotective mechanisms and cellular.

Ouchi et al. [52] state that obesity is related to an increase in inflammatory markers which characterized a low-grade chronic inflammation. In this study, we noted that HFD provokes increase in inflammatory markers. However, the treatment with EEA (200- 400mg/kg/bw) showed a significant decrease in inflammatory markers. The anti-inflammatory properties of* Cynara* were confirmed by Ben Salem et al. [36] in the report of nonalcoholic fatty liver disease.

Vial et al. [53] found a relationship between HFD and increased reactive oxygen species (ROS) production in the liver. ROS causes cell damage via the mechanism implying lipid peroxidation and protein oxidation that disposes tissue damage, especially in the liver [54]. In the current investigation, we found that HFD caused oxidative stress in the liver as revealed by increasing in ROS levels production as mentioned in Table 7, accompanied by increasing in lipid peroxidation and decreasing in antioxidant activities. Ohara et al. [55] reported that HFD was a set of chronic pathologies that can cause hypercholesterolemia and oxidative stress.

Among the antioxidants systems, antioxidant enzymes such as GSH, SOD, and GPx present great defense mechanisms, which play a pivotal role in maintaining the ROS under adequate concentrations. In the current study, EEA (200-400 mg/kg/bw) treatment provokes an increase of GSH, SOD, and GPx activities. As similar results found in Dianita et al. [16] and Abdel Majid et al. [34] studies, it has also been reported that* Cynara* leaves extract treatment caused significant increases in GSH-Px activities in the liver [56]. Kuçükgergin et al. [40] reported that* Cynara *leaves extract caused significant decreases in MDA levels in the liver tissues with an increase in hepatic GSH-Px activities in hypercholesterolemic rats. Moreover, Ben Salem et al. [11] proved the antioxidative effect of EEA (200-400 mg/kg bw) in the liver tissues of the diabetic rat.

The report of Mehmetcik et al. [56] showed a decrease in hepatic MDA and conjugated dienes levels and an increase in GSH and GSH-Px activities after treatment with* Cynara *leaves extract. An increase of antioxidant activity in erythrocytes was also found by Küçükgergin et al. [40], after treatment with water extract from* Cynara *which confirmed the antioxidant properties of this plant.

Many* in vitro* studies have shown that the antioxidant potential of* Cynara *is dependent on radical scavenging and metal ion chelating effect of its active constituents such as cynarin, chlorogenic acid, and flavonoids [46, 57]. Nevertheless, the* in vivo* efficiency has not been confirmed sufficiently. Most* in vivo* studies correlated to the antioxidative capacity of* Cynara *and the hepatoprotective effect [58].

## 6. Conclusion

*Cynara scolymus* leaves extracts, especially their ethanol extract, exert a potential effect on rats induced by a high fat-rich diet. The administration of antioxidant-rich* Cynara *extract ameliorates the undesirable effect of a high fat diet on lipid accumulation and hepatic disorders.

## Figures and Tables

**Table 1:** Identification of phenolic compounds in ethanol extract from leaves of *C. scolymus* using their retention times, LC/MS, and LC–MS/MS data.

Peak^a^	RT (min)	m/z	Mode (+/-)	*ƛ*_max_ (min)	MS/MS	Tentative ID
1	3.00	122.3	+	200-700	82	Salicylic acid-o-hexoside
2	3.7	117.2	-	200-700	100	Chlorogenic acid
3	4.2	112	-	200-700	214.8, 178.8, 352.8	3-mono-o-cafeoylquinic acid
4	5.3	141	-	200-700	265.9	Apigenin-7-glucoside
5	8.4	223	-	200-700	331.4	Unknown
6	10.30	275	-	200-700	529.7, 350.8, 284.7	Luteolin-7-o-rutinoside
7	13.5	327.1	-	200-700	170.9	Dihydroxypropiophend-hexoside
8	14.8	395	-	200-700	529.7, 288. 9,307	Kampferol 3-o-rutinoside
9	16.2	431	-	200-700	434.7, 294.9, 344.8	Quercetin-o-pentoside
10	23.3	623	-	200-700	255.1	Caffeic acid

Identification was aided by comparison with reference standards that were available and by correlation with previous literature reports.

^a^ Peak numbers and retention times (Rt) refer to LC/MS chromatograms in Figure 2.

**Table 2:** The inhibitory capacity of *C. scolymus* leaves extracts against lipase pancreatic activity.

	Concentration (ug/ml)	% Inhibition	CI50 (ug/ml)
Orlistat	25	80.25 ± 1.08	17.76
	50	97.21 ± 0.59	
	100	90.35 ± 1.20	

Hexane	25	17.46 ± 0.05	200.15
	50	20.13 ± 1.19	
	100	49.81 ± 0.28	

Ethylacetate	25	-	-
	50		
	100		

Butanol	25	23.09 ± 1.88	101.50
	50	30.13± 1.02	
	100	70.18 ± 0.07	

Ethanol	25	60.38 ± 1.58	61.50
	50	69.23 ± 0.84	
	100	73.90 ± 1.77	

Aqueous	25	36.81 ± 0.07	95.78
	50	62.10 ± 1.02	
	100	67.24 ± 0.13	

All data are expressed as mean ± SD. (-): inactive.

**Table 3:** Effect of treatment with *C. scolymus* leaves extract on body weight.

Groups	Cont	HFD	HFD+EEA (200mg/kg/bw)	HFD+EEA (400mg/kg/bw)	HFD+ATOR
Day 0	210.00 ± 0.67	265.66 ± 3.45*∗∗∗*	260.45 ± 2.21	266.00 ± 2.13	255.78 ± 2.30*∗∗*
Day 65	214.50±10.40	280.10±13.93*∗∗∗*	222.5 ± 15.00^###^	234.33 ±10.11^###¥^	216.66 ±18.00^###^

HFD groups were orally administered with ethanol extract (EEA) from leaves of *C. scolymus* and atorvastatin (ATOR) at the doses mentioned earlier for 60 days. Values are given as means ± SD (n = 6).*∗∗* p ≤ 0.01 and *∗∗∗* p ≤ 0.001 were considered significant compared to control groups; ^###^ p ≤ 0.001 was considered significant compared to HFD groups; ^¥^p ≤ 0.05 was considered significant compared to HFD groups treated with ATOR.

**Table 4:** Effect of treatment with *C. scolymus* leaves extract on serum lipid profile.

Groups	Cont	HFD	HFD+ EEA (200mg/kg / bw)	HFD+EEA (400mg/kg/bw)	HFD+ATOR
TG (mmol/L)	1.00 ± 0.29	2.50±0.40*∗∗∗*	1.13 ± 0.20^###^	0.8± 0.42^###^	1.00 ± 0.29^###^
T-Ch (mmol/L)	1.37 ± 0.12	3.50±0.55*∗∗∗*	1.60 ± 0.15^###^	1.70 ± 0.15^###^	1.62 ± 0.08^###^
LDL- c (mmol/L)	0.60 ± 0.19	1.80±0.07*∗∗∗*	1.30± 0.35^###^	0.80 ± 0.01^###^	0.45 ± 0.40^###^
HDL-c (mmol/L)	1.44 ± 0.33	0.50±0.10*∗∗∗*	0.80 ± 0.33^###^	1.19± 0.07^###¥^	0.76 ± 0.08^###^
AIP	0.95 ± 0.17	6.88±0.14*∗∗∗*	2.00 ± 0.12^###^	1.48 ±0.19^###¥¥^	2.13 ± 0.20^###^
CRI-I	0.951	7	2	1.42	2.13

HFD groups were orally administered with ethanol extract (EEA) from leaves of *C. scolymus* and atorvastatin (ATOR) at the doses mentioned earlier for 60 days. Values are given as means ± SD (n = 6). *∗∗∗* p ≤ 0.001 was considered significant compared to control groups; ^###^ p ≤ 0.001 was considered significant compared to HFD groups; ^¥^p ≤ 0.05 and ^¥¥^ p ≤ 0.01 were considered significant compared to HFD groups treated with ATOR.

**Table 5:** Effect of treatment with* C. scolymus* leaves extract on liver dysfunction enzymes.

Groups	AST (UI/L)	ALT (UI/L)	LDH (UI/L)	ALP (UI/L)	OCT (mg / g protein)
Cont	131.9 ± 1.50	42.76 ± 1.78	76.2 ± 2.5	64.78 ± 2.50	10.5±1.3
HFD	273.7 ±2.33*∗∗∗*	76.03±7.31*∗∗∗*	137±8.03*∗∗∗*	206.66± 6.52*∗∗∗*	7.9±0.5*∗*
HFD+EEA(200mg/kg/bw)	128.56 ±7.87^###^	41.51 ± 8.03^###^	70.18 ±8.03^##^	72.83 ± 8.00^##^	5.7±0.2^#^
HFD+EEA(400mg/kg/bw)	112.18 ±5.47^###^	36.56 ±1.85^###¥^	63.2 ±1.85^###^	72.20 ± 3.00^##^	6.0±0.1
HFD+ATOR	169.18 ±4.37^###^	49.36±3.73^###¥¥^	84.5 ± 1.22^##^	89.2 ± 4.18^##^	6.4±0.7

HFD groups were orally given ethanol extract (EEA) from leaves of *C. scolymus* and atorvastatin (ATOR) at the doses mentioned earlier for 60 days. Values are given as means ± SD (n = 6). *∗*p ≤ 0.05 and *∗∗∗* p ≤ 0.001 were considered significant compared to control groups; ^#^ p ≤ 0.05, ^##^ p ≤ 0.01, and ^###^ p ≤ 0.001 were considered significant compared to HFD groups; ^¥^p ≤ 0.05 and ^¥¥^ p ≤ 0.01 were considered significant compared to HFD groups treated with ATOR.

**Table 6:** Effect of treatment with* C. scolymus* leaves extracts on hematological parameters.

Groups	Cont	HFD	HFD+EEA (200mg/kg/bw)	HFD+EEA (400mg/kg/bw)	HFD+ATOR
GR (×10^6^)	6.96 ± 2.37	9.13 ± 0.09	8.91 ± 0.04^¥¥^	9.00 ± 0.07^#¥¥¥^	8.48 ± 0.04^###^
Hb (g/dL)	12.55 ± 0.91	15.15± 0.91*∗∗∗*	14.85± 0.63	15.00 ± 0.21	14.80 ± 0.14
HCT(fL)	36.25± 0.96	44.35 ± 1.90*∗∗∗*	43.45 ± 1.90	38.55± 0.35^#¥¥¥^	35.75 ± 0.07^#^
VGM(fL)	50.55 ± 0.63	54.9±3.53	48.75 ± 2.61	50.9 ± 0.00	50.55 ± 0.63
TCMH (g/dL)	13.45± 4.73	33.7± 0.15*∗∗*	16.65 ± 0.91^##^	16.75 ± 0.07^###¥¥¥^	17.45 ± 0.07^##^
MCHC (g/dL)	31.6± 1.97	33.7± 1.27	30.2 ± 0.00	32.85 ± 0.21	32.85 ± 0.21
GB (×10^3^ /uL)	6.82 ± 2.06	17.45 ± 2.04*∗∗*	10.41 ± 2.06^#^	11.2 ±1.12^#^	9.46 ±1.08^##^
PLT (×10^3^/uL)	859 ± 9.23	1260 ± 9.34*∗∗∗*	839.5± 8.45^##^	914.5± 11.87^##^	879 ± 8.21^##^

HFD groups were orally administered with ethanol extract (EEA) from leaves of *C. scolymus* and atorvastatin (ATOR) at the doses mentioned earlier for 60 days. Values are given as means ± SD (n = 6). *∗∗* p ≤ 0.01 and *∗∗∗* p ≤ 0.001 were considered significant compared to control groups; ^#^ p ≤ 0.05, ^##^ p ≤ 0.01, and ^###^ p ≤ 0.001 were considered significant compared to HFD groups; ^¥¥^ p ≤ 0.01 and ^¥¥¥^ p ≤ 0.001 were considered significant compared to HFD groups treated with ATOR.

**Table 7:** Effect of treatment with* C. scolymus* leaves extract on liver oxidative stress parameters.

Groups	Liver ROS (fluorescence intensity, FI)	TBARS (nmol MDA/ mg protein)	AOPP (nmol/mg protein)	SOD (U SOD/mg protein)	GSH (ug/mg protein)	GPx (nmol GSH min/mg protein)
Cont	70.02 ±11.23	8.64 ± 0.43	4.62 ± 0.01	3.79±0.31	5.45 ± 0.41	6.46 ± 0.60
HFD	150.30±20.34*∗∗∗*	18.15±0.92*∗∗∗*	8.85±0.08*∗∗∗*	1.91±0.04*∗∗∗*	2.06 ± 0.14*∗∗∗*	4.55 ± 0.28*∗∗∗*
HFD+EEA(200mg/kg/bw)	80.23±15.17^##^	10.51± 0.56^###^	5.12 ± 0.05^###^	2.84±0.19^###^	4.17± 0.28^###¥¥¥^	5.81 ± 0.17^###^
HFD+EEA(400mg/kg/bw)	84.17± 16.57^###^	9.25 ± 0.61^###^	4.53± 0.04^###^	2.91±0.04^###^	5.10 ± 0.15^###^	6.00 ± 0.56^###¥^
HFD+ATOR	72.49±13.45^###^	10.44 ±1.03^###^	4.37 ± 0.05^###^	2.49±0.49^###^	4.09 ± 0.33^###^	5.45 ± 0.15^###^

HFD groups were orally administered with ethanol extract (EEA) from leaves of *C. scolymus* and atorvastatin (ATOR) at the doses mentioned earlier for 60 days. Values are given as means ± SD (n = 6). *∗∗∗* p ≤ 0.001 was considered significant compared to control groups; ^###^ p ≤ 0.001 was considered significant compared to HFD groups; ^¥^ p ≤ 0.05 and ^¥¥¥^ p ≤ 0.001 were considered significant compared to HFD groups treated with

ATOR.

## Data Availability

The data used to support the findings of this study are available from the corresponding author upon request.
